# Experimental Investigation on the Performance Degradation of TPO Waterproofing Membranes Under Coupled Thermo-Oxidative Aging and Freeze–Thaw Cycles

**DOI:** 10.3390/polym18161944

**Published:** 2026-08-08

**Authors:** Jinsen Wang, Fangfang Wang, Jicheng Sun, Qingyang Li, Qi Ren, Guojun Sun

**Affiliations:** 1 China Electronics Engineering Design Institute Co., Ltd., Beijing 100142, China; wangjinsen@sdic.com.cn (J.W.); renqi@ceedi.cn (Q.R.); 2 Beijing General Municipal Engineering Design & Research Institute Co., Ltd., Beijing 100761, China; 15117912996@163.com; 3 Baoding Construction Engineering Tendering and Bidding Service Center, Baoding 071000, China; sunjicheng5@163.com; 4 Faculty of Architecture, Civil and Transportation Engineering, Beijing University of Technology, Beijing 100124, China

**Keywords:** TPO waterproofing membrane, coupled aging, low–high-temperature cycling, Fourier-transform infrared spectroscopy, peel resistance, tensile response

## Abstract

Thermoplastic polyolefin (TPO) waterproofing membranes are widely used in building roofs and underground waterproofing systems, and their durability under complex service environments is critical to long-term waterproofing reliability. Self-adhesive TPO membranes from one production batch were evaluated in the unaged condition (C0) and after 5, 7, 14, and 28 complete coupled aging cycles (C5, C7, C14, and C28, respectively). Each complete cycle lasted 48 h; thus, C5, C7, C14, and C28 corresponded to total elapsed times of 10, 14, 28, and 56 d. Mass variation, Fourier-transform infrared spectroscopy (FTIR), peel, tensile, and scanning electron microscopy (SEM) measurements were integrated to evaluate physical, near-surface chemical, interfacial, and mechanical changes. The reported mass-loss ratios varied only slightly from 0.25% to 0.35%, indicating limited sensitivity of mass variation under the adopted conditions. FTIR band-depth indices near 1021, 876, 1462, and 719 cm^−1^ changed non-monotonically with cycle number, whereas SEM images showed a visually more heterogeneous exposed TPO surface after aging. At C28, the mean peak peel resistance decreased from 94.2 ± 3.1 to 28.5 ± 0.6 N/50 mm (69.8%), while the mean nominal tensile stress at 250 mm crosshead extension decreased from 2.59 ± 0.14 to 2.06 ± 0.05 MPa (20.7%). No tensile specimen ruptured within the investigated extension range. These descriptive results indicate that the self-adhesive joint response was more sensitive to the adopted coupled aging protocol than the fixed-extension tensile response of the membrane.

## 1. Introduction

Waterproofing membranes are essential functional materials used in roofs, underground spaces, and construction details to resist water ingress, and their long-term service performance is directly associated with the durability of building envelopes, structural service safety, and maintenance costs. In practical engineering environments, waterproofing membranes are continuously exposed to complex environmental factors, including elevated temperature, oxygen, moisture, ultraviolet radiation, and temperature cycling. Under these conditions, material degradation generally initiates from changes in the surface chemical structure and deterioration of the micro-morphology, and subsequently develops into reduced interfacial bonding capacity and deterioration of macroscopic mechanical properties. Therefore, durability evaluation of waterproofing membranes should not be limited to initial performance indicators, but should also focus on the intrinsic relationships among chemical structure, micro-morphology, and macroscopic mechanical response during aging. Wang et al. [1] investigated the performance evolution of SBS-modified asphalt waterproofing membranes under the combined effects of thermo-oxidative aging and freeze–thaw cycling. Their results showed that the coupled aging environment reduced the low-temperature flexibility and tensile performance of the membranes, while Fourier-transform infrared spectroscopy (FTIR) was effective in revealing changes in chemical structure. Li et al. [2] further compared the mechanical properties, low-temperature adaptability, and interlayer compatibility of different waterproofing membranes after thermo-oxidative aging, and reported significant differences in the sensitivity of different material systems to thermo-oxidative environments. These studies indicate that establishing the relationship between aging behavior and performance degradation mechanisms is essential for accurately evaluating the service durability of waterproofing membranes.

The aging of polymeric waterproofing materials is generally associated with oxidation of polymer chains, migration of low-molecular-weight components, depletion of stabilizers, and evolution of surface microstructures. Klempova et al. [3] investigated the structural changes in plasticized polyvinyl chloride (PVC-P) after thermal and ultraviolet aging using UV–Vis spectroscopy, ATR-FTIR, and Raman spectroscopy. Their results demonstrated that spectroscopic analysis can effectively identify characteristic chemical changes during the aging of PVC-P materials. Martins et al. [4] examined the surface, rheological, and morphological characteristics of isotactic polypropylene microplastics exposed to photo-thermo-oxidative conditions, and found that such aging markedly altered the surface state and structural response of polyolefin materials. Novotny [5] reported that, for plasticized PVC waterproofing membranes, plasticizer migration is a key factor affecting service life and the feasibility of subsequent repair. Kravanja et al. [6] investigated the deterioration characteristics of aged plasticized PVC waterproofing membranes through mechanical testing, wettability measurements, scanning electron microscopy (SEM), FTIR, and thermal analysis. They found that plasticizer loss led to surface hardening, increased porosity, and subsequent degradation of mechanical properties. These studies provide an important basis for understanding the aging mechanisms of polymeric waterproofing membranes; however, most existing investigations have focused on PVC, PVC-P, or polypropylene systems, whose aging behavior and damage mechanisms cannot be directly generalized to TPO waterproofing membranes.

To more realistically reproduce engineering service environments, durability studies on waterproofing materials have gradually shifted from single-factor aging tests to multi-factor coupled or scenario-based aging simulations. Tan et al. [7] investigated the performance evolution of polyester-reinforced self-adhesive asphalt waterproofing membranes subjected to thermo-oxidative aging and freeze–thaw cycling. They found that aging significantly reduced the low-temperature flexibility and peel strength of the material, accompanied by oxidation reactions and the loss of light components. Sitzia et al. [8] simulated daily variations, seasonal hygrothermal conditions, and solar radiation effects using building stone as the research object. Their results indicated that, compared with purely extreme conditions, aging protocols closer to real climatic processes are more helpful for interpreting service-induced damage mechanisms. Waldvogel et al. [9] investigated the crack-bridging behavior of one-component cementitious flexible waterproofing membranes and showed that temperature and crack-opening rate significantly affected their deformation mechanism and crack-bridging capacity. Zhou et al. [10] investigated the low-temperature flexibility and peel strength of self-adhesive polymer-modified bituminous waterproofing membranes after heat aging and found that thermal aging adversely affected both properties. These findings indicate that aging evaluation of waterproofing materials should fully consider the coupled relationships among environmental actions, material composition, and testing indicators, because a single performance index is insufficient to comprehensively characterize the service degradation process.

Long-term durability prediction and post-service performance assessment are also important aspects of waterproofing membrane research. Ivanic and Lubej [11] investigated PVC-P roofing waterproofing membranes after 11 years of service and found that conventional mechanical indicators may not always sufficiently reveal the actual degree of material deterioration. In contrast, dynamic fatigue testing, SEM, and EDS analyses were more sensitive in identifying the causes of material failure. Kovac et al. [12] examined changes in the physical and mechanical properties of roofing waterproofing materials under ultraviolet radiation, humidity, and temperature, and pointed out that these properties directly affect the service life of waterproofing materials and the effectiveness of subsequent repair. Luciani et al. [13] evaluated the long-term durability of PVC-P waterproofing geomembranes through laboratory tests and extrapolated their service life based on plasticizer loss and changes in mechanical properties. Subsequently, Luciani et al. [14] further investigated the long-term durability and service-life prediction of PVC waterproofing membranes, emphasizing that accelerated aging protocols should approximate actual service environments as closely as possible. Francke et al. [15] studied the mechanical properties of flexible waterproofing membranes used on ventilated terraces after long-term exposure to elevated temperatures, and found that high-temperature exposure altered the load-bearing capacity and impact resistance of the materials. Wang et al. [16] investigated the humidity response of polymer-modified cementitious waterproofing membranes prepared with different polymer emulsions, showing that material composition significantly affects stability in humid environments. Collectively, these studies have deepened the understanding of waterproofing material durability from the perspectives of service-life prediction, environmental response, and performance retention.

In addition to the deterioration of the intrinsic material properties, the interfacial bonding performance and repairability after aging also determine whether waterproofing systems can continue to serve reliably. Garrido et al. [17] investigated the repair performance of aged PVC waterproofing membranes and compared the effects of hot-air welding and solvent-based adhesive bonding on the shear and peel properties between new and aged membranes. Their results showed that different joining methods significantly affected the mechanical reliability of the repaired interface. Zhang et al. [18] studied the aging-dependent repair performance and interfacial durability of new–aged waterproofing membrane systems. They found that the degree of aging affected peel strength, low-temperature flexibility, and interfacial morphology, thereby influencing the long-term reliability of repaired systems. These studies indicate that aging of waterproofing membranes not only reduces the intrinsic strength of the material, but also changes the mechanical response of lap joints, welded joints, or repaired interfaces. For TPO waterproofing membranes, which are increasingly used in engineering applications, peel performance and tensile performance reflect the interfacial bonding capacity and intrinsic load-bearing capacity of the membrane, respectively. Therefore, simultaneous evaluation of these two properties is essential for assessing the service risk of aged TPO waterproofing membranes.

Although previous studies have provided valuable insights into accelerated aging of PVC/PVC-P, asphalt-based, cementitious, and other polymeric waterproofing membranes, the degradation behavior of self-adhesive TPO membranes under sequential thermo-oxidative and low–high-temperature cycling remains insufficiently understood. TPO membranes differ from plasticized PVC and asphalt-based membranes in polymer composition, additive system, surface characteristics, and interfacial bonding behavior; therefore, degradation patterns reported for those materials cannot be transferred directly to self-adhesive TPO systems. In particular, limited information is available on the relationships among near-surface chemical changes, exposed-surface morphology, self-adhesive joint degradation, and fixed-extension tensile response under the same coupled aging history.

To address these gaps, self-adhesive TPO waterproofing membranes from the same production batch were subjected to a sequential coupled-aging procedure consisting of thermo-oxidative exposure and low–high-temperature cycling under coolant immersion. Mass variation, ATR–FTIR, SEM, peel, and tensile measurements were integrated within the same experimental framework to characterize physical, near-surface chemical, exposed-surface morphological, interfacial, and fixed-extension mechanical changes.

The main advances of this study over previous investigations are threefold. First, the self-adhesive TPO membrane and its membrane-to-membrane bonded interface were evaluated under the same coupled aging history, rather than treating material aging and interfacial performance as separate issues. Second, the FTIR and SEM results were considered together with peel and tensile responses to establish qualitative relationships between near-surface changes and macroscopic performance degradation. Third, the aging sensitivity of the bonded interface was directly compared with that of the TPO membrane matrix, while an early-stage characterization point was included to identify initial changes in mass, surface chemical structure, and micro-morphology. This integrated approach provides experimental evidence for identifying aging-sensitive indicators and assessing the durability and maintenance requirements of self-adhesive TPO waterproofing systems.

## 2. Experimental Section

### 2.1. Test Materials

**Figure 1:**
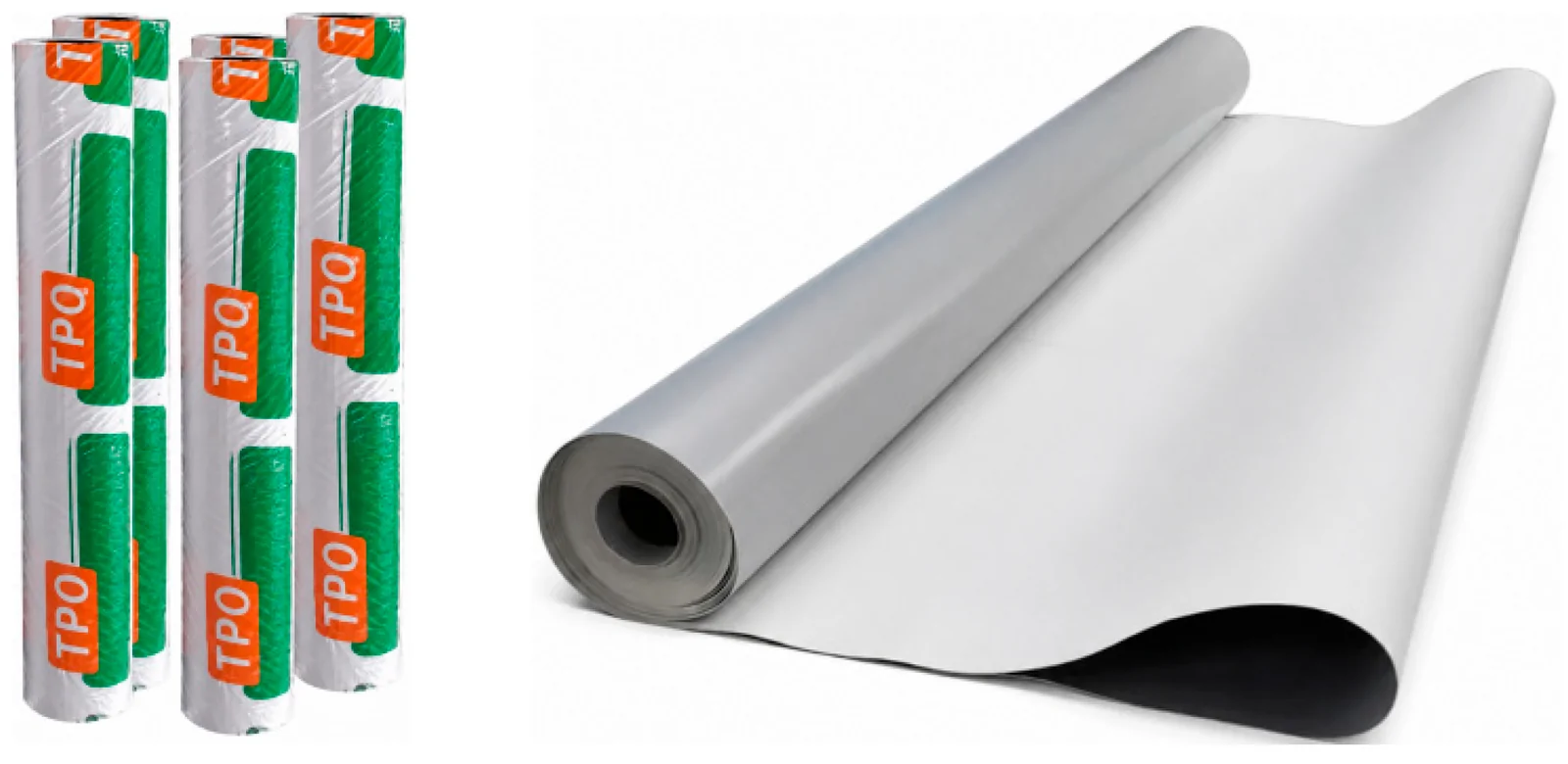
TPO polymer waterproofing membrane.

The test material was a commercial self-adhesive thermoplastic polyolefin (TPO) waterproofing membrane supplied by Oriental Yuhong Co., Ltd. (Beijing, China), as shown in Figure 1. The product consisted of a non-reinforced homogeneous H-type TPO sheet and a factory-applied synthetic polymer adhesive layer, without an internal fabric reinforcement layer or fleece backing. According to the manufacturer’s published technical information, the TPO sheet was based primarily on thermoplastic elastomeric polymers derived from α-olefins, such as ethylene and propylene. The formulation also contained functional components, including flame retardants, light-screening agents, antioxidants, and light stabilizers.

Before specimen preparation, the membrane thickness was measured in accordance with GB/T 328.5-2007 [19] at five uniformly distributed positions; the mean measured thickness was 1.50 mm. To minimize batch, edge, and cutting-direction effects, all specimens were cut from the central region of the same membrane roll, with the long axis parallel to the machine direction. Tensile specimens were rectangular strips 200 mm long and 50 mm wide. Each peel specimen comprised two strips, each 100 mm long and 50 mm wide; the detailed bonded geometry is described in Section 2.3. For FTIR and SEM, the analyzed surface was the exterior exposed TPO sheet surface, opposite the factory-applied self-adhesive layer. It was not the adhesive bonding surface used in the peel specimens. The unaged specimens served as the C0 controls.

### 2.2. Aging Scheme

**Figure 2:**
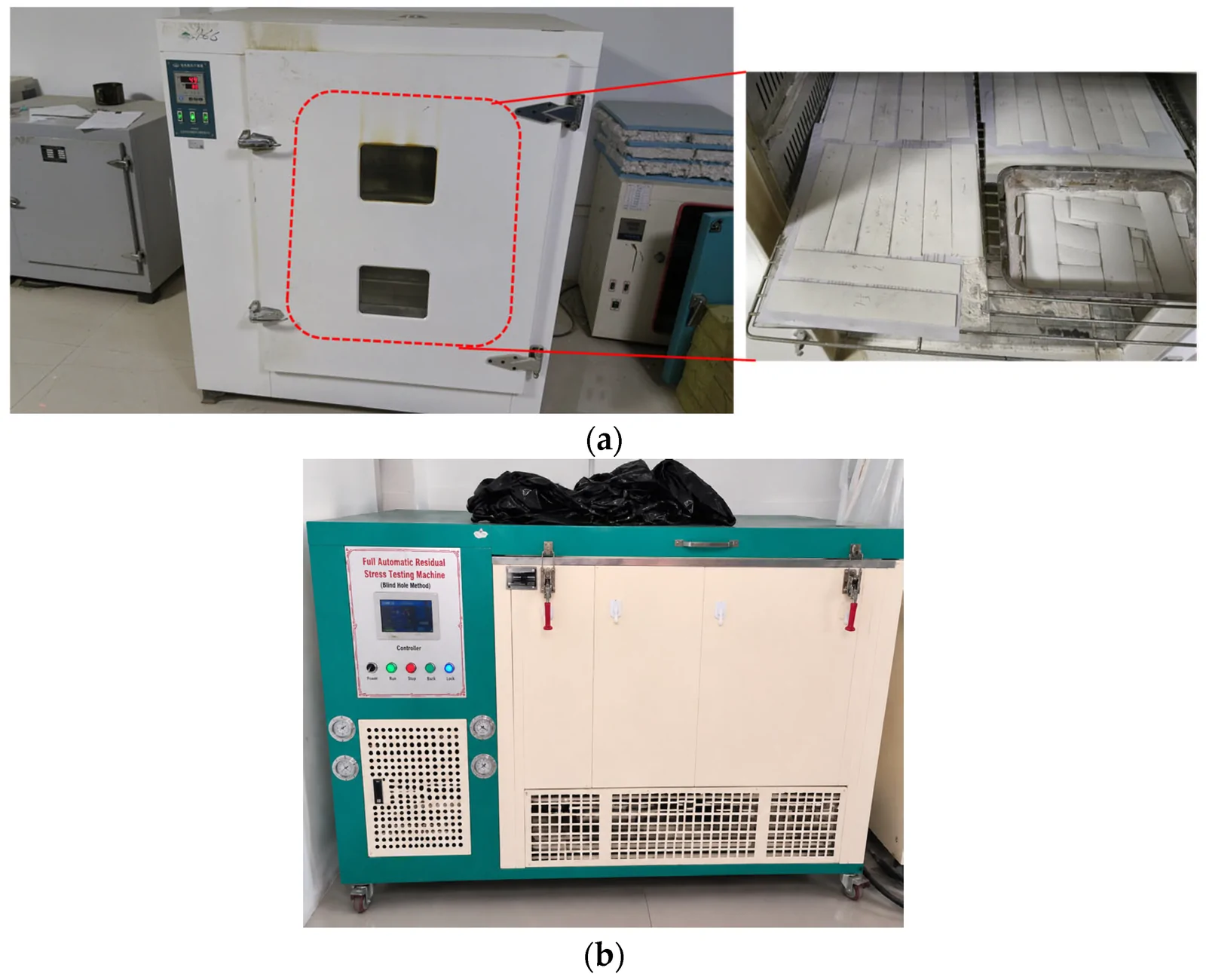
Aging test equipment: (**a**) hot-air aging chamber; (**b**) high–low-temperature freeze–thaw cycling chamber.

To investigate the performance evolution of TPO waterproofing membranes under coupled thermo-oxidative and low–high-temperature actions, a two-stage accelerated aging procedure was adopted. The aging temperatures were selected to represent severe thermal conditions encountered by waterproofing membranes in building-envelope applications. Under intense summer solar radiation, roof and exterior-wall interface temperatures may rise substantially; therefore, 80 °C was adopted as the thermo-oxidative temperature with reference to reported high-temperature measurements [20]. In the second stage, a programmed temperature range from −20 to +20 °C was applied while the specimens were immersed in a commercial low-temperature-resistant coolant. This protocol provided a controlled accelerated condition for comparing degradation under sequential thermal and low–high-temperature actions, rather than a direct simulation of a specific outdoor service period. The test setup is shown in Figure 2.

One complete coupled aging cycle lasted 48 h and consisted of two consecutive stages. First, specimens were held in a hot-air aging chamber at 80 °C for 24 h. They were then transferred to the high–low-temperature chamber and immersed in a commercial low-temperature-resistant coolant for another 24 h, comprising 12 h at −20 °C followed by 12 h at +20 °C. The experimental conditions were coded C0, C5, C7, C14, and C28, where the numeral denotes the number of complete 48-h coupled cycles. The corresponding total elapsed times were 0, 10, 14, 28, and 56 d, respectively. This distinction between cycle number and elapsed time was used throughout the manuscript.

After completion of the prescribed aging cycles, all specimens were removed from the coolant, surface-dried, and conditioned at 23 °C for 24 h. Mass, FTIR, and SEM measurements were performed for C0, C5, C7, C14, and C28. Peel and tensile tests were performed for C0, C7, C14, and C28. C5 was retained as an early characterization point for physical, near-surface chemical, and surface-morphological observations, but mechanical tests were not performed for C5.

### 2.3. Test Methods

To evaluate the effects of coupled environmental aging on the physical properties, chemical structure, interfacial bonding performance, and micro-morphology of TPO waterproofing membranes, mass variation, Fourier-transform infrared spectroscopy (FTIR), peel, tensile, and scanning electron microscopy (SEM) tests were conducted.

The mass of each specimen was measured using an electronic balance before and after aging, and mass variation was evaluated for each defined aging condition. Five independent specimens were measured per condition.

ATR–FTIR spectra were acquired using a Bruker INVENIO-S Fourier-transform infrared spectrometer equipped with a Platinum diamond ATR accessory and an RT-DLaTGS detector (Bruker Optics GmbH & Co. KG, Ettlingen, Germany). The exterior exposed TPO sheet surface, opposite the self-adhesive layer, was used for all measurements. Spectra were recorded in transmittance mode over the range of 4000–400 cm^−1^ at a spectral resolution of 4 cm^−1^, using 16 co-added sample scans and 16 background scans. The spectra were retained in transmittance form for presentation. No multiplicative intensity normalization was applied to the full spectra, and vertical offsets were used only for visual clarity. For the complementary integrated band-area analysis described below, the transmittance spectra were converted to absorbance according to $A=-\log_{10}T$. To quantitatively characterize the variations in the selected absorption bands, a relative peak-depth index was calculated from the transmittance spectra. For each target absorption band b under aging condition *t*, the actual peak position was identified as the wavenumber corresponding to the minimum transmittance within a predefined search window W*_b_*.

$${\tilde{\nu }}_{b,t}^{*}=\arg\underset{\tilde{\nu }\in W_{b}}{\min}T_{t}(\tilde{\nu })$$

where *b* denotes the target absorption band, t denotes the aging condition, $\tilde{\nu }$ is the wavenumber, W*_b_* is the predefined wavenumber search window for band b, $T_{t}(\tilde{\nu })$ is the transmittance spectrum under aging condition t, and ${\tilde{\nu }}_{b,t}^{*}$ is the detected peak position of band *b*.

A local linear baseline $B_{b,t}({\tilde{\nu }}_{b,t}^{*})$ was constructed using the two shoulder regions surrounding each target absorption band. The baseline-corrected peak depth was calculated as

$$D_{b,t}=B_{b,t}^{T}({\tilde{\nu }}_{b,t}^{*})-T_{t}({\tilde{\nu }}_{b,t}^{*})$$

where $B_{b,t}^{T}({\tilde{\nu }}_{b,t}^{*})$ is the baseline transmittance at the detected peak position, and $D_{b,t}$ is the baseline-corrected peak depth of band b under aging condition t.

The relative peak-depth index was then defined as

$$R_{b,t}=\frac{D_{b,t}}{D_{b,0}}$$

where *D_b_*_,0_ is the baseline-corrected peak depth of the corresponding absorption band in the unaged specimen, *R_b_*,_*t*_ is the relative peak-depth index, and the subscript 0 denotes the unaged condition. Accordingly, *R_b_*_,0_ = 1. A value of *R_b_*,_*t*_ > 1 indicates that the corresponding absorption band became deeper than that of the unaged specimen, whereas *R_b_*,_*t*_ < 1 indicates that the band became shallower. The relative peak-depth index is dimensionless.

The predefined search windows were 1435–1495, 1000–1055, 850–900, and 700–740 cm^−1^ for the absorption bands near 1462, 1021, 876, and 719 cm^−1^, respectively. The values obtained from the peak-depth analysis were normalized to the baseline-corrected peak depth of the corresponding absorption band in the unaged specimen. As a complementary semi-quantitative analysis, baseline-corrected integrated band areas were also calculated from the absorbance spectra. For each target band *b* under aging condition *t*, the same predefined wavenumber window *W_b_* and local linear-baseline procedure used in the peak-depth analysis were adopted. The baseline-corrected integrated band area was calculated as

$$S_{b,t}=\int_{W_{b}}\max\left[A_{t}(\tilde{v})-B_{b,t}^{A}(\tilde{v}),0\right]d\tilde{v}$$

where $A_{t}(\tilde{\nu })$ is the absorbance spectrum, $B_{b,t}^{A}(\tilde{\nu })$ is the local linear absorbance baseline, and *S_b_*,_*t*_ is the baseline-corrected integrated area of band *b*. The integrated CH-stretching envelope over 3000–2800 cm^−1^ was used as the internal reference. The normalized band-area index was defined as

$$I_{b,t}^{\mathrm{area}}=\frac{S_{b,t}/S_{\mathrm{CH},t}}{S_{b,0}/S_{\mathrm{CH},0}}$$

where *S_CH_*,_*t*_ is the baseline-corrected integrated area over 3000–2800 cm^−1^ and the subscript 0 denotes the unaged condition. Accordingly, $I_{b,t}^{\mathrm{area}}$ = 1, and the normalized band-area index is dimensionless.

For each aging condition, one specimen was analyzed by FTIR. The 16 co-added scans used to generate each spectrum were instrumental signal averages rather than independent experimental replicates. Therefore, the FTIR results were used for descriptive and semi-quantitative comparison among the different aging conditions.

Peel tests were conducted on membrane-to-membrane self-adhesive joints with reference to GB/T 328.21-2007 [21]. No rigid substrate was used. Each joint comprised two membrane strips, each 100 mm long and 50 mm wide. After the release films had been removed, the two factory-prepared self-adhesive surfaces were brought into contact over a bonded length of 50 mm, producing a bonded area of 50 mm × 50 mm and leaving an unbonded 50-mm free gripping end on each strip. No additional liquid adhesive, adhesive tape, or other bonding agent was used. The joints were prepared and pressed before aging; the complete bonded specimens were then subjected to the prescribed coupled aging treatment.

After the prescribed aging treatment, peel specimens were conditioned at 23 °C for 24 h. The two unbonded free ends were mounted in opposing grips. The initial distance between grips was 100 ± 5 mm, and testing was conducted at 23 ± 2 °C with a grip-separation speed of 100 mm/min. The force–displacement response and failure morphology were recorded continuously until the joint was completely separated. Because every specimen was 50 mm wide, the measured force was reported as peel resistance in N/50 mm. Three independent joint specimens were tested for each mechanical aging condition.

Tensile tests were conducted on rectangular specimens 200 mm long and 50 mm wide with reference to GB/T 328.9-2007 [22]. Five independent specimens were tested per mechanical aging condition. The nominal initial grip separation recorded for the test setup was 200 ± 2 mm, and the crosshead speed was 100 mm/min. Because the stated specimen length and nominal grip separation were equal, the resulting crosshead-based force–extension response was treated as a comparative fixed-extension index rather than as a standardized ultimate tensile property. Reference marks were used to monitor grip slippage. Tests were continued until crosshead extension exceeded 250 mm and were then manually terminated within the safe operating range; no grip slippage was observed.

For quantitative comparison, the tensile force at a common extension of 250 mm, *F*_250_, was extracted from each force–extension curve by linear interpolation between the two adjacent recorded data points. The corresponding nominal tensile stress was calculated as

$$\sigma_{250}=\frac{F_{250}}{bh}$$

where b = 50 mm is the specimen width and h = 1.50 mm is the measured mean membrane thickness. A crosshead extension of 250 mm corresponds to a nominal engineering strain of 125% relative to the recorded initial grip separation. Because extension was obtained from crosshead displacement, and because the specimen length equaled the nominal grip separation, this strain and the associated stress are apparent, fixed-extension comparison indices. Ultimate tensile strength, elongation at break, and Young’s modulus were not determined.

Surface micro-morphology of the exterior exposed TPO sheet surface, opposite the self-adhesive layer, was observed using a SEM5000Pro field-emission scanning electron microscope (CIQTEK Co., Ltd., Hefei, China). Specimens were mounted on stubs with conductive carbon tape and sputter-coated with gold for 90 s. Observations were conducted at 10.0 kV and ×1000 magnification. For each aging condition, at least three non-overlapping fields on the same analyzed specimen were examined, and one representative micrograph was selected for subsequent presentation. The images were compared qualitatively in terms of surface uniformity, particle-like features, local agglomerations, groove-like textures, and localized irregularities; they were not used for quantitative roughness or defect-density measurements.

Data analysis was descriptive. Mass measurements used five independent specimens per condition; peel and tensile tests used three and five independent specimens per mechanical condition, respectively. In the graphical presentation of the mechanical test results, thin curves show individual specimens and the bold black curve shows the group mean. Shaded bands around mean curves and endpoint error bars denote mean ±1 standard deviation (SD), while colored symbols show individual endpoint values. Because the replicate numbers were limited and all specimens came from a single production batch and aging program, no inferential hypothesis tests or *p* values were used; between-condition differences are interpreted descriptively.

## 3. Results and Discussion

### 3.1. Mass Variation

**Figure 3:**
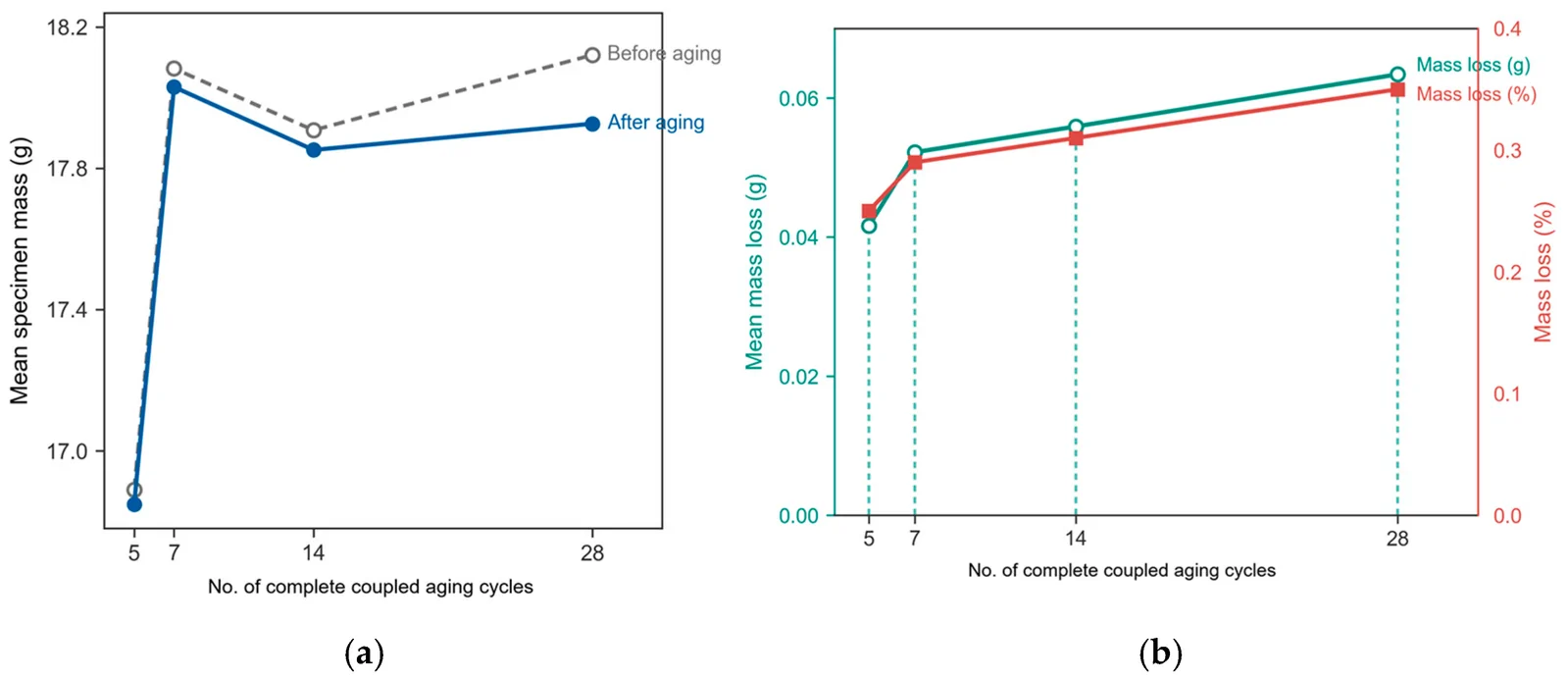
Mass variation of TPO waterproofing membranes at the investigated coupled-aging conditions: (**a**) average mass before and after aging; (**b**) average mass loss and mass-loss ratio.

The mass variation of TPO waterproofing membranes at different coupled-aging conditions is presented in Figure 3 and Table 1. The results indicate that coupled aging caused a continuous but limited mass loss in the TPO waterproofing membranes. At C5, C7, C14, and C28, the average mass losses were 0.0416, 0.0522, 0.0559, and 0.0634 g, respectively, corresponding to average mass loss ratios of 0.25%, 0.29%, 0.31%, and 0.35%. Although the initial average mass differed among aging groups, resulting in slight fluctuations in the absolute mass values, the normalized mass loss ratio showed a clear increasing trend with cycle number. The mass loss ratio remained below 0.40% across the investigated conditions, suggesting that the TPO membrane maintained relatively good mass stability under the adopted coupled aging conditions. No abrupt mass reduction or distinct stage-wise deterioration was observed, indicating that mass variation mainly occurred as a slow and continuous material loss process. The small but continuous mass loss indicates a limited net loss of material during aging. The specific components contributing to this mass change were not resolved in the present study. In actual roof service environments, ultraviolet radiation may further accelerate surface oxidation, stabilizer depletion, and microstructural deterioration, thereby intensifying the aging process of TPO waterproofing membranes. However, the limited mass loss observed in this study also indicates that mass variation alone is not a highly sensitive indicator for characterizing early aging damage.

### 3.2. FTIR Analysis

**Figure 4:**
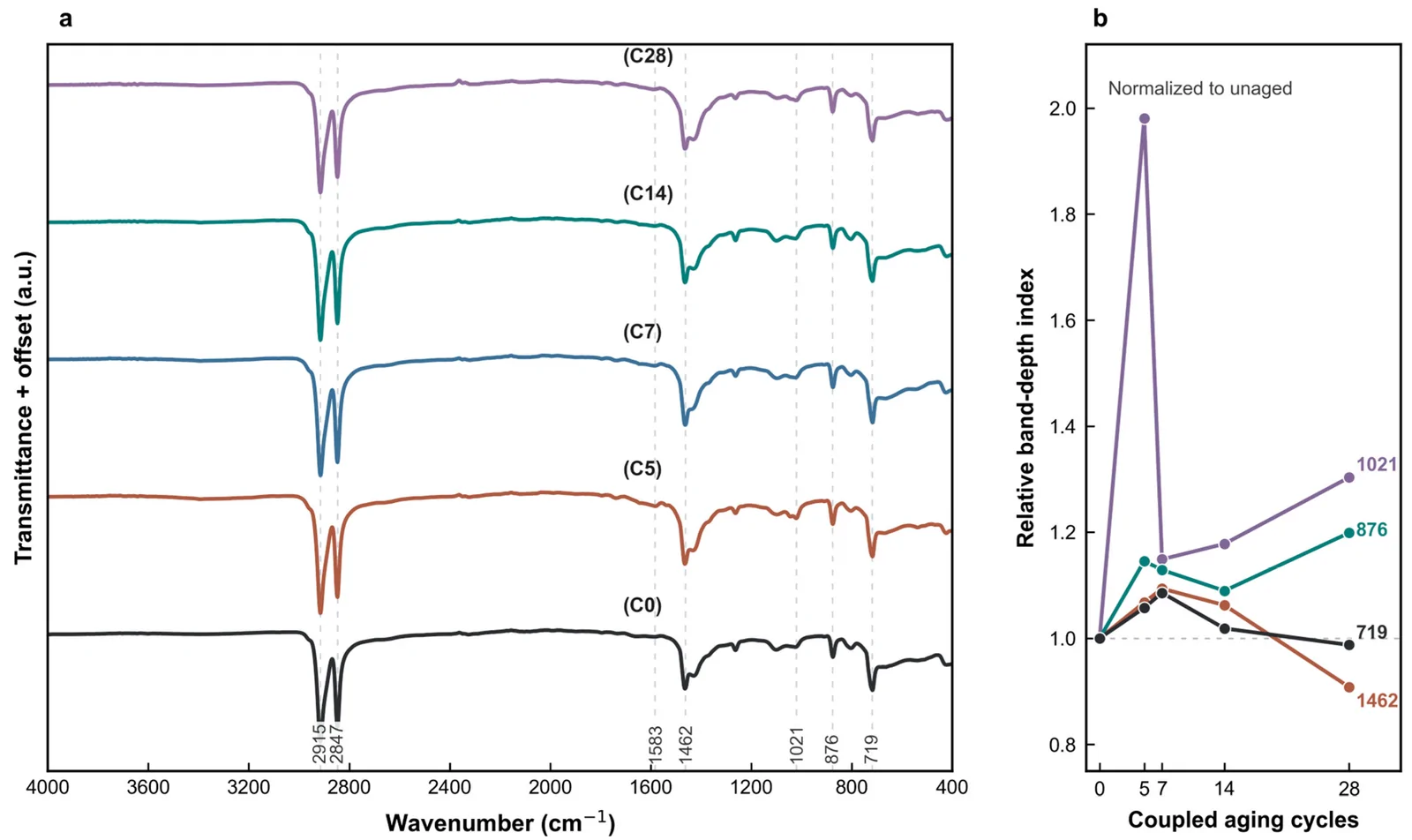
FTIR spectra and evolution of relative peak-depth indices of TPO waterproofing membranes at C0, C5, C7, C14, and C28: (**a**) FTIR spectra in the range of 4000–400 cm^−1^; (**b**) evolution of relative band-depth indices for the characteristic absorption bands.

As shown in Figure 4a, all specimens exhibited distinct absorption bands near 2915 and 2847 cm^−1^, which are mainly attributed to the stretching vibrations of -CH_2_- groups in the polyolefin matrix. The absorption band near 1462 cm^−1^ is generally associated with the bending vibration of -CH_2_- groups, whereas the band near 719 cm^−1^ can be assigned to the rocking vibration of long-chain methylene sequences. After coupled aging, changes were also observed in the bands near 1021 and 876 cm^−1^. Previous FTIR studies on talc-containing polyolefin membranes have attributed the absorption band near 1018–1021 cm^−1^ to Si-O-Si stretching vibrations of talc or related silicate components [23]. In addition, the band near 876 cm^−1^ is a characteristic absorption band of calcite-type calcium carbonate and is associated with the out-of-plane bending vibration of carbonate groups [24]. Therefore, the bands near 1021 and 876 cm^−1^ in the present membrane may be tentatively associated with silicate- and carbonate-based filler components, respectively.

Because the commercial membrane contained fillers and functional additives, the band assignments should be regarded as tentative. Variations near 1021 and 876 cm^−1^ are interpreted as changes in the relative near-surface spectral contribution of silicate- and carbonate-related components, respectively. No monotonic increase was observed in the 1800–1650 cm^−1^ carbonyl region. This may reflect limited net oxidation within the ATR sampling depth, formulation-dependent stabilization, band overlap, or near-surface heterogeneity; the absence of a monotonic carbonyl increase does not demonstrate an absence of aging. Previous heat-aging work on formulated TPO films has likewise combined FTIR with thermal and mechanical methods to distinguish formulation-dependent responses [25]. Complementary surface-compositional and thermal analyses would be needed to resolve the chemical origin.

The relative peak-depth index near 1021 cm^−1^ increased at C5, decreased at C7 and C14, and increased again at C28. The index near 876 cm^−1^ varied modestly at intermediate conditions and reached approximately 1.20 at C28. The 1462 cm^−1^ index at C28 was lower than that at C0, while the 719 cm^−1^ index increased slightly at early conditions and later approached the C0 level. As shown in Table 2, the baseline-corrected integrated band-area indices, normalized to the CH-stretching envelope and to C0, provide a complementary semi-quantitative characterization of these spectral variations. These band-specific, non-monotonic variations indicate that the near-surface FTIR response did not evolve uniformly with cycle number. Because only one independently aged specimen was analyzed per condition, the observations are treated as descriptive and semi-quantitative rather than inferential.

### 3.3. Peel Resistance

**Figure 5:**
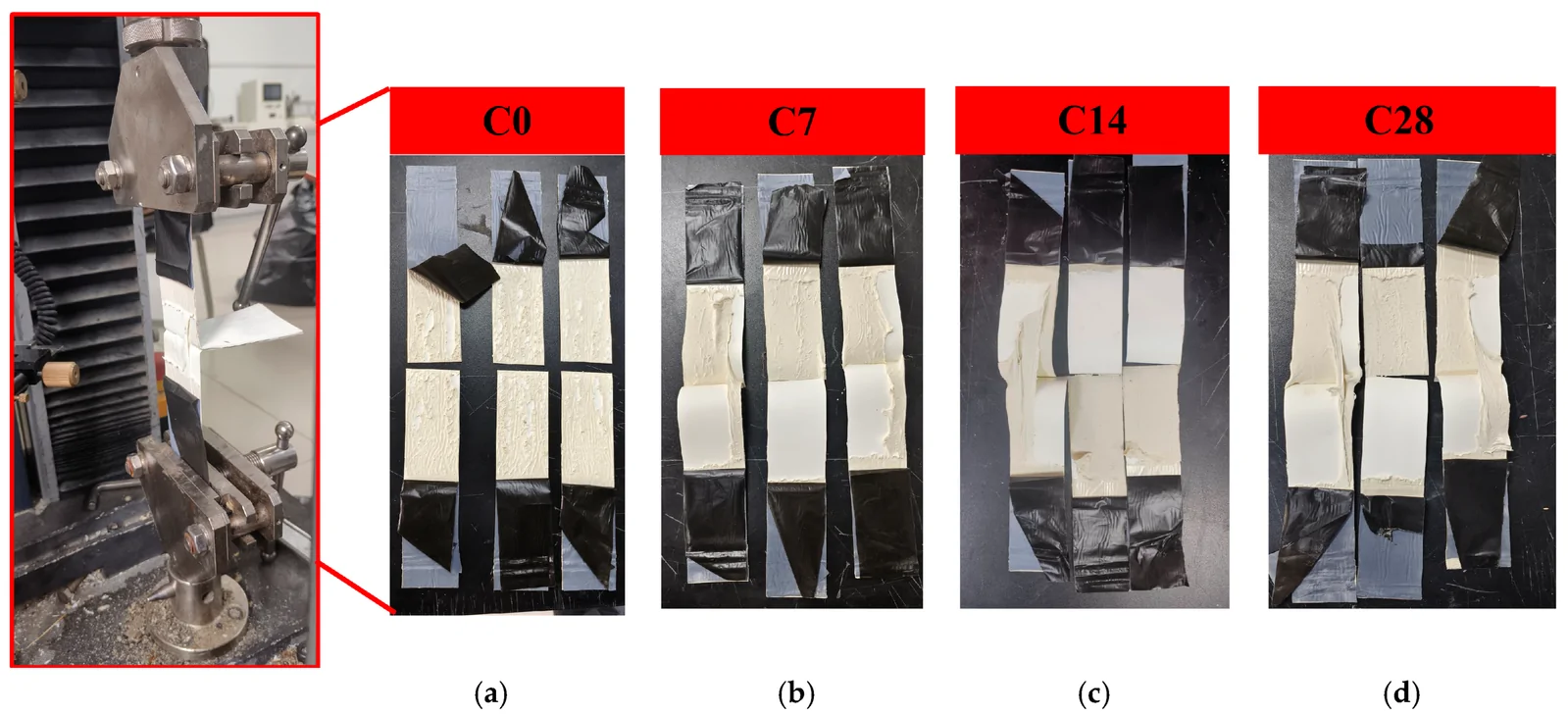
Peel failure morphologies of the self-adhesive joints at (**a**) C0, (**b**) C7, (**c**) C14, and (**d**) C28.

Figure 5 shows the peel failure morphologies at C0, C7, C14, and C28. At C0, residual self-adhesive material was distributed relatively continuously over the opposing adhesive surfaces. At C7, the separated joint showed more non-uniform residue, local curling, and tearing. At C14 and C28, the residue distribution and peel path appeared increasingly irregular, with regions of comparatively smooth debonding. These observations indicate a transition from relatively stable separation toward more spatially heterogeneous adhesive/interfacial and localized cohesive failure. The comparisons are qualitative and do not constitute a quantitative classification of failure-mode fractions.

**Figure 6:**
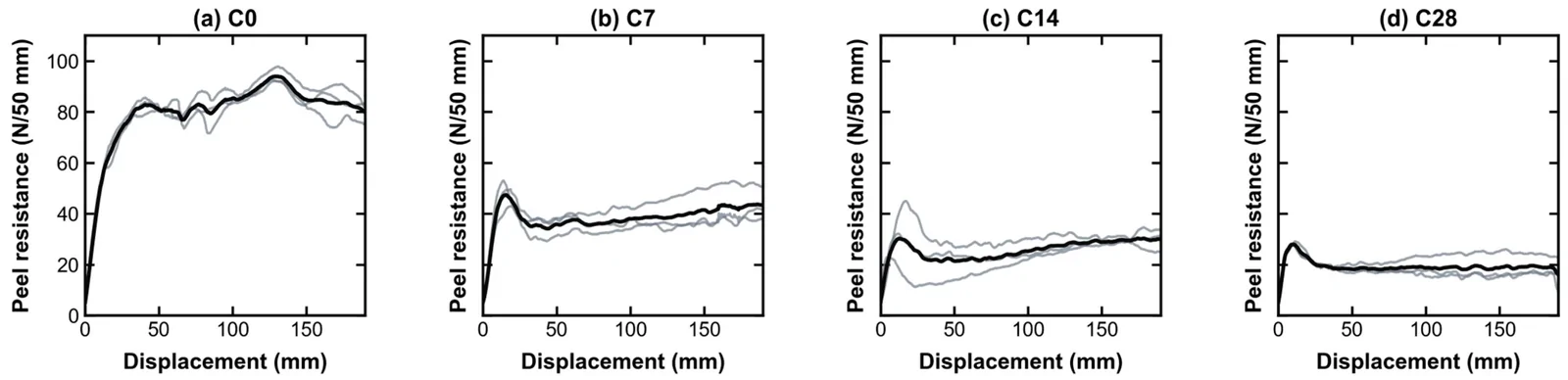
Peel-resistance–displacement curves at (**a**) C0, (**b**) C7, (**c**) C14, and (**d**) C28. Thin gray curves represent three independent joint specimens, and the bold black curve is the group mean.

As shown in Figure 6, the peel-resistance–displacement curves exhibited a progressive reduction in peak peel resistance with increasing aging cycles. The mean peak peel resistance decreased from 94.2 ± 3.1 N/50 mm at C0 to 50.1 ± 4.9, 36.2 ± 7.6, and 28.5 ± 0.6 N/50 mm at C7, C14, and C28, respectively (*n* = 3). The C0-to-C28 reduction was 69.8%. These values are descriptive group statistics. The marked reduction is consistent with the increasingly heterogeneous failure morphologies, although the present experiment does not separate the individual contributions of thermal exposure and low–high-temperature cycling.

**Figure 7:**
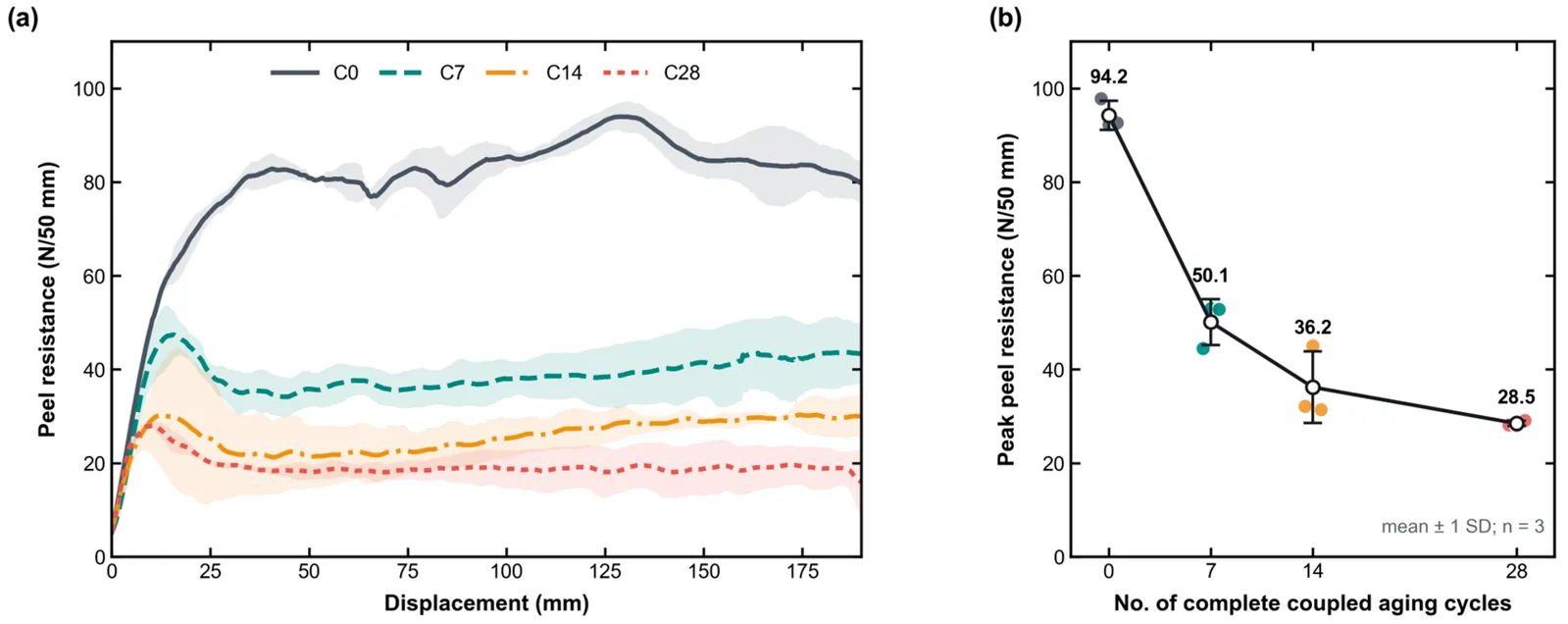
Peel response under coupled aging: (**a**) group mean peel-resistance–displacement curves with shaded bands denoting ±1 SD (*n* = 3); (**b**) peak peel resistance, with colored points showing individual specimens and open symbols with error bars showing mean ±1 SD. Cx denotes x complete 48-h coupled aging cycles.

As shown in Figure 7, the C0 group maintained the highest peel-resistance plateau. The plateau decreased markedly at C7 and decreased further at C14 and C28, while the aged curves also showed greater local fluctuation. This behavior is compatible with increasingly intermittent crack propagation and local debonding during peeling. Because the FTIR and SEM measurements were performed on the exterior TPO sheet surface rather than on the self-adhesive bonding surface, those observations cannot be used as direct evidence of changes at the adhesive-to-adhesive interface. The mechanical and surface observations therefore provide complementary, but not causally conclusive, evidence of aging-related changes. From the perspective of engineering maintenance, even when the TPO membrane retains a certain tensile load-bearing capacity, the reduction in peel performance of the aged surface may become the controlling factor for the failure of seams, laps, or local repair regions.

### 3.4. Tensile Response

**Figure 8:**
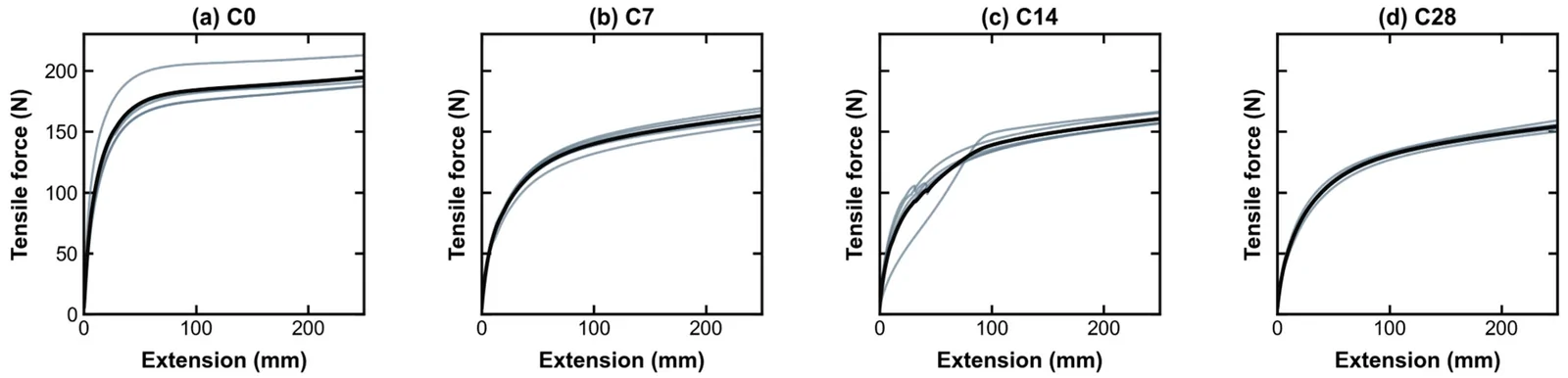
Tensile force–extension curves at (**a**) C0, (**b**) C7, (**c**) C14, and (**d**) C28. Thin gray curves represent five independent specimens, and the bold black curve is the group mean.

The tensile response reflects resistance to large in-plane deformation under the recorded test geometry. Figure 8 shows force–extension curves for five independent specimens in each mechanical aging condition. Over the common 0–250 mm extension range, all specimens exhibited a nonlinear response: force increased rapidly at first and then increased more gradually. No abrupt force drop occurred within this range, consistent with the absence of rupture.

The C0 force–extension curves were generally above the aged curves over most of the investigated range. A marked downward shift was present at C7. The C7, C14, and C28 curves were comparatively close, with a smaller subsequent decrease as cycle number increased. These descriptive results indicate that the largest observed change in the fixed-extension tensile response occurred between C0 and the first mechanically tested aged condition, C7; no tensile data were collected at C5.

All 20 tensile specimens reached 250 mm crosshead extension, and the minimum terminal extension was 252.4 mm. None ruptured or slipped in the grips. Curve endpoints therefore represent manual test termination rather than failure. The results characterize a crosshead-based large-deformation response and must not be interpreted as ultimate tensile strength or elongation at break.

**Figure 9:**
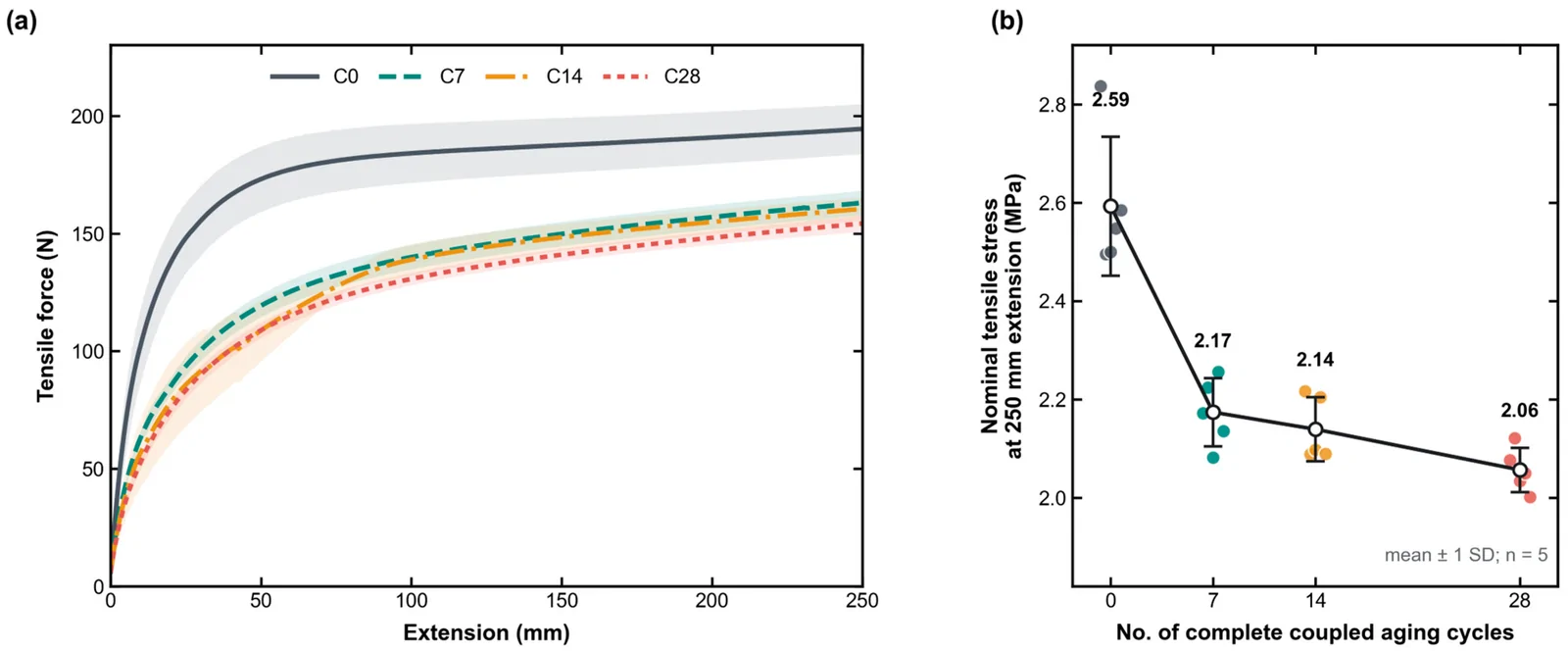
Tensile response under coupled aging: (**a**) group mean force–extension curves with shaded bands denoting ±1 SD (*n* = 5); (**b**) nominal tensile stress at 250 mm extension, with colored points showing individual specimens and open symbols with error bars showing mean ±1 SD. Cx denotes x complete 48-h coupled aging cycles.

To compare the different aging groups at the same deformation level, the nominal tensile stress was calculated at an extension of 250 mm, corresponding to 125% nominal engineering strain. As shown in Figure 9, the mean nominal tensile stresses were 2.59 ± 0.14, 2.17 ± 0.07, 2.14 ± 0.07, and 2.06 ± 0.05 MPa for the 0, 7, 14, and 28 d groups, respectively (*n* = 5). The value decreased by 16.2% during the first 7 d and by 20.7% after 28 d. The relatively small differences between the 7, 14, and 28 d groups are consistent with the close spacing of their force–extension curves.

The downward shift in the aged force–extension curves and the reduction in nominal tensile stress at a fixed deformation indicate that coupled aging reduced the resistance of the membrane to large tensile deformation. This reduction may be associated with aging-induced changes in the polymer matrix and the progressive development of surface defects observed by FTIR and SEM [3,4]. However, the 20.7% reduction in tensile response was considerably smaller than the 69.9% reduction in peel resistance. This comparison indicates that the self-adhesive interface was more sensitive to coupled aging than the bulk tensile response of the membrane. Because ultimate tensile failure was not reached and no extensometer was used, the present results do not provide ultimate tensile strength, elongation at break, or Young’s modulus.

### 3.5. Surface Micro-Morphology

**Figure 10:**
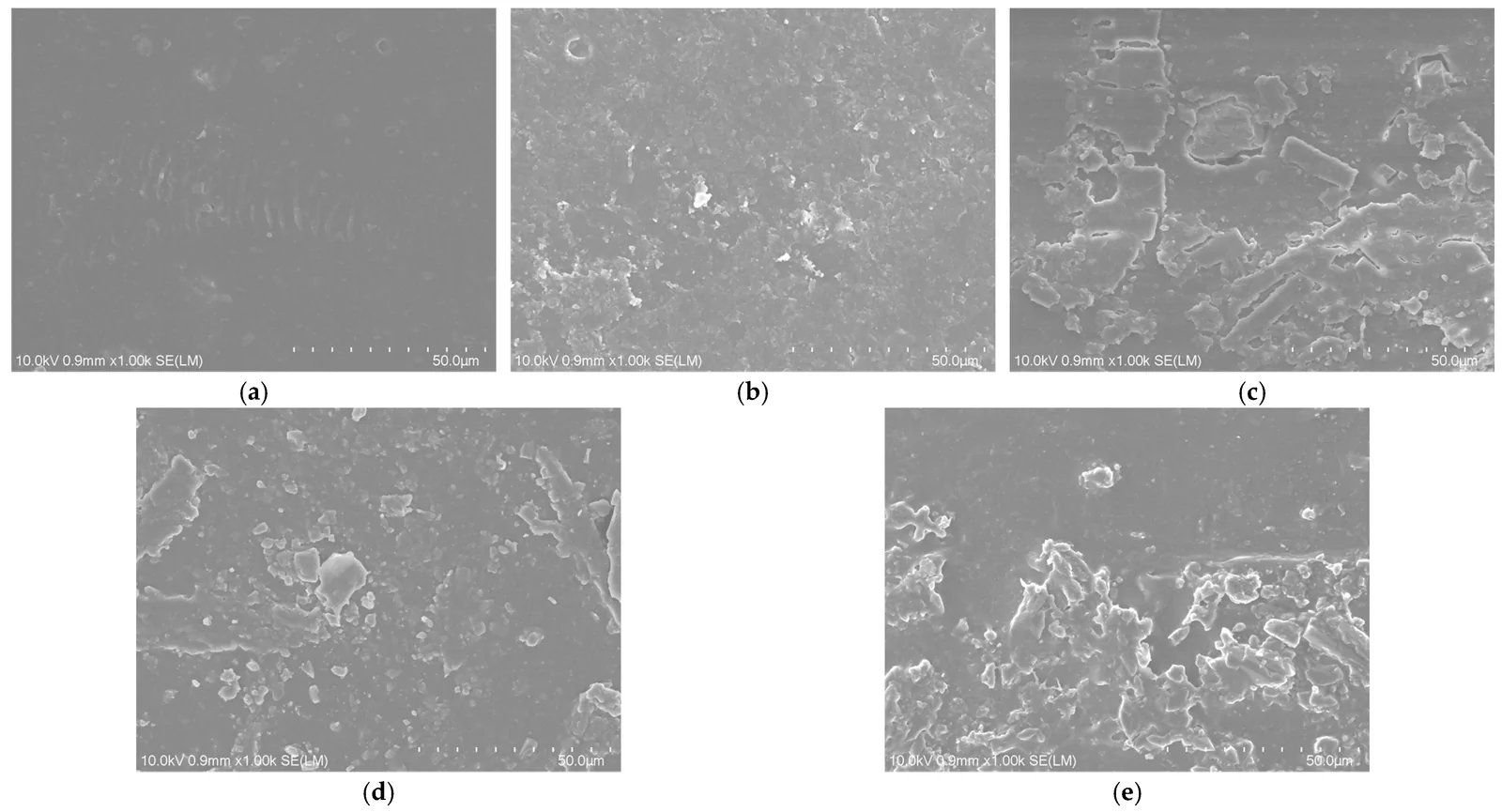
Exterior exposed TPO surface micrographs under coupled aging: (**a**) C0, (**b**) C5, (**c**) C7, (**d**) C14, and (**e**) C28 (×1000; scale bar = 50 μm).

The surface micro-morphologies observed by SEM are shown in Figure 10. The unaged specimen exhibited a relatively smooth surface with few visible defects, indicating good continuity of the surface layer. After 5 coupled aging cycles, scattered particle-like features and slight surface unevenness began to appear. With further aging, the surface became progressively rougher and more heterogeneous, accompanied by increasingly evident local agglomerations, groove-like textures, and localized defects. After 28 coupled aging cycles (C28), these features were more widely distributed, indicating a progressive deterioration in surface integrity and uniformity.

The qualitative SEM observations and mechanical changes were obtained under the same sequence of coupled aging conditions, but they represent different surfaces and response scales. FTIR and SEM characterized the exterior TPO sheet surface, whereas peeling occurred between two factory-applied self-adhesive surfaces. The main polyolefin bands remained visible, and the reported mass-loss ratio was small. By contrast, the mean peak peel resistance and fixed-extension nominal tensile stress decreased by 69.8% and 20.7%, respectively, from C0 to C28. The evolution of the exterior surface morphology occurred alongside the reduction in peel resistance. Surface irregularities, local agglomerations, and defect-like features, if present at the bonded interface, could reduce contact uniformity and promote local stress concentrations during peeling. However, the exterior-surface observations cannot directly demonstrate that this mechanism governed adhesive-joint deterioration.

Thermo-oxidative exposure alone has been reported to reduce mechanical performance and interlayer compatibility in related waterproofing membranes [2]. Sequential thermo-oxidative and freeze–thaw aging also reduced tensile or peel performance in SBS-modified [1] and polyester-based self-adhesive asphalt membranes [7]. The decreases observed in the present TPO membrane are consistent with this general deterioration trend. Heat-aged TPO films have also exhibited formulation-dependent spectral and physical changes, with the onset of deterioration varying according to the stabilizer formulation [24]. However, all specimens in the present study were obtained from one production batch, and no thermo-oxidative-only or low–high-temperature-only control groups were included. Therefore, the separate contributions and interaction of the two aging stages cannot be resolved. Within these limitations, the present membrane showed a more pronounced loss of adhesive-joint peel resistance than of fixed-extension tensile resistance. Differences in the magnitude and distribution of degradation among the reported membrane systems may be related to the polymer matrix, reinforcement structure, adhesive-interface configuration, stabilizer and filler formulation, and exposure conditions.

## 4. Conclusions

Based on mass variation, FTIR, peel, tensile, and SEM results of TPO waterproofing membranes after different coupled aging durations, this study analyzed the effects of aging on bulk stability, surface chemical structure, interfacial bonding capacity, and surface micro-morphology. The main conclusions are as follows.

(1) The TPO waterproofing membrane exhibited continuous but limited mass loss during aging. After aging for 5, 7, 14, and 28 d, the average mass loss ratios were 0.25%, 0.29%, 0.31%, and 0.35%, respectively. This indicates that the membrane maintained good overall mass stability, although mass variation showed limited sensitivity to aging damage.
(2) FTIR results showed that the characteristic bands associated with the polyolefin backbone remained evident after aging. However, the relative intensities of the bands near 1021, 876, 1462, and 719 cm^−1^ changed, indicating that aging mainly affected the surface chemical environment, and reveal an aging-dependent evolution of the near-surface spectral characteristics of the membrane.
(3) Coupled aging markedly reduced both the peel resistance of the self-adhesive joints and the nominal tensile response of the TPO membrane. After 28 d of aging, the mean peak peel resistance decreased from approximately 94.2 to 28.5 N/50 mm, representing a reduction of 69.8%. At a nominal engineering strain of 125%, the mean nominal tensile stress decreased from 2.59 ± 0.14 to 2.06 ± 0.05 MPa, corresponding to a reduction of 20.7%. None of the tensile specimens ruptured within the investigated displacement range, indicating that the membrane retained substantial deformation capacity despite the decrease in tensile stress at the prescribed deformation level.
(4) SEM observations showed that the membrane surface gradually evolved from a relatively smooth morphology to a more heterogeneous and irregular state after aging, accompanied by particle aggregation, localized pores, and other surface defects. These qualitative morphological changes were consistent with the FTIR spectral variations and the reduction in peel resistance, collectively indicating aging-related deterioration of the membrane’s near-surface condition. Therefore, a comprehensive durability assessment of TPO membranes should consider mass variation, spectroscopic characteristics, surface micro-morphology, and mechanical performance.

## Figures and Tables

**Table 1:** Reported mass-loss results at the investigated coupled-aging conditions.

Aging Condition	Average Mass Loss/g	Average Mass Loss Ratio/%
C5	0.0416	0.25
C7	0.0522	0.29
C14	0.0559	0.31
C28	0.0634	0.35

**Table 2:** Baseline-corrected integrated band-area indices normalized to the CH-stretching envelope and to C0.

Aging Condition	1462 cm^−1^	1021 cm^−1^	876 cm^−1^	719 cm^−1^
C0	1.00	1.00	1.00	1.00
C5	1.04	1.65	1.02	0.91
C7	1.04	1.03	0.99	0.90
C14	0.96	1.08	0.95	0.91
C28	1.02	1.25	1.20	0.99

## Data Availability

Data are contained within the article.
